# Rumination symptoms in treatment-resistant major depressive disorder, and outcomes of repetitive Transcranial Magnetic Stimulation (rTMS) treatment

**DOI:** 10.1038/s41398-023-02566-4

**Published:** 2023-09-08

**Authors:** Stephanie A. Chu, Reza Tadayonnejad, Juliana Corlier, Andrew C. Wilson, Cole Citrenbaum, Andrew F. Leuchter

**Affiliations:** 1 Neuroscience Interdepartmental Program, UCLA Los Angeles, USA; 2 TMS Clinical and Research Service, Neuromodulation Division, Semel Institute for Neuroscience and Human Behavior at UCLA, Los Angeles, CA USA; 3 Department of Psychiatry and Biobehavioral Sciences, David Geffen School of Medicine at UCLA, Los Angeles, CA USA; 4 Division of the Humanities and Social Sciences, California Institute of Technology, Pasadena, CA USA

**Keywords:** Depression, Human behaviour

## Abstract

Rumination is a maladaptive style of regulating thoughts and emotions. It is a common symptom of Major Depressive Disorder (MDD), and more severe rumination is associated with poorer medication and psychotherapy treatment outcomes, particularly among women. It is unclear to what extent rumination may influence the outcomes of, or be responsive to, repetitive Transcranial Magnetic Stimulation (rTMS) treatment of MDD. We retrospectively examined data collected during rTMS treatment of 155 patients (age 42.52 ± 14.22, 79 female) with moderately severe treatment-resistant MDD. The severity of rumination and depression was assessed before and during a course of 30 sessions of measurement-based rTMS treatment using the Ruminative Responses Scale (RSS) and the Patient Health Questionnaire (PHQ-9), respectively. Relationships among baseline levels of rumination, depression, and treatment outcome were assessed using a series of repeated measures linear mixed effects models. Both depression and rumination symptoms significantly improved after treatment, but improvement in depression was not a significant mediator of rumination improvement. Higher baseline rumination (but not depression severity) was associated with poorer depression outcomes independently of depression severity. Female gender was a significant predictor of worse outcomes for all RRS subscales. Both depressive and ruminative symptoms in MDD improved following rTMS treatment. These improvements were correlated, but improvement in rumination was not fully explained by reduction in depressive symptoms. These findings suggest that while improvement in rumination and depression severity during rTMS treatment are correlated, they are partly independent processes. Future studies should examine whether rumination symptoms should be specifically targeted with different rTMS treatment parameters.

## Introduction

Rumination is a maladaptive pattern of regulating thoughts and emotions characterized by a repetitive focus on negative thoughts such as dwelling on negative memories and analyzing events without taking action [1]. It is a transdiagnostic behavioral element, as defined by the National Institute of Mental Health’s Research Domain Criteria (RDoC), associated with vulnerability to a number of neuropsychiatric disorders [2, 3]. Rumination is most strongly linked to depression, increasing the length and severity of episodes, increasing the likelihood of relapse, and exacerbating negative moods [1], primarily among women [4]. Furthermore, it amplifies negative thoughts and impairs problem-solving behavior, decreasing the motivation of depressed patients to seek solutions [5].

It is not clear whether rumination represents an enduring trait or a treatable symptom in patients with major depressive disorder (MDD). Some data indicate that rumination is an enduring “response style” that confers “trait vulnerability” to episodes of depression [3]. While behavioral interventions including mindfulness meditation and rumination-focused cognitive behavioral therapy may reduce ruminative symptoms [3, 6, 7], the evidence is mixed on whether rumination is responsive to pharmacotherapy. Ketamine has been shown to reduce ruminative symptoms in treatment-resistant depression and reduce negative self-focus in healthy controls [8, 9]. One study of depressed adolescents, however, found that medication alone did not reduce ruminative symptoms [10]. A randomized controlled trial showed that in patients with medication-refractory depression, rumination severity decreased only when their treatment-as-usual antidepressant use was coupled with rumination-focused cognitive behavioral therapy, suggesting that antidepressant medication alone is not enough to reduce rumination [7], although another study found that antidepressant use significantly reduced rumination [11]. Overall, the literature suggests that rumination is a malleable state, albeit one that is difficult to ameliorate with antidepressants alone.

Rumination is a prominent feature in many patients with MDD [1, 12] that has been associated with poorer medication and psychotherapy treatment outcomes [13, 14]. The patient cohorts in these previous studies had mild-to-moderate depression so it remains unclear how rumination may influence treatment outcome among those with more severe, treatment-refractory depression. It is also unclear whether rumination severity influences the outcome of repetitive Transcranial Magnetic Stimulation (rTMS) treatment for MDD. One prior study has shown that rumination can be ameliorated with rTMS [15], an increasingly common, effective, and safe treatment method for treatment-refractory MDD [16, 17].

In the present study, we sought to confirm and extend earlier results by examining the relationships among baseline rumination levels, depression severity, and rTMS treatment outcome in a cohort of treatment-resistant MDD patients. We hypothesized that rTMS treatment would help reduce rumination symptoms in MDD, but that severity of rumination would have a negative relationship with rTMS outcome.

## Methods

### Subjects

This is a retrospective study of patients treated for MDD from 2020 to 2023 by the UCLA TMS Clinical and Research Service. The sample consisted of 204 participants with a primary diagnosis of MDD confirmed by the Mini International Neuropsychiatric Interview (MINI) [18]. All subjects had treatment-resistant MDD as indicated by a lifetime history of four or more failed antidepressant trials (due to a lack of response or tolerability). Data on the specific medications, duration of illness, and hospitalizations were not available for these subjects. We include here only those subjects who completed the Ruminative Responses Scale (RRS) of the Response Styles Questionnaire [1] and Patient Health Questionnaire (PHQ-9) [19] at the baseline (treatment 1) and final visits (treatment 30), yielding a final cohort of 155 patients. All patients underwent at least 30 rTMS treatment sessions. Most of the patients received medication in conjunction with their rTMS treatment. This retrospective analysis of deidentified data was approved by the UCLA Institutional Review Board (IRB).

### Primary clinical measures

The 22-item RRS was collected to assess rumination, and the 9-item PHQ-9 was collected to assess depression severity at seven time points over the course of rTMS treatment: baseline, and treatments 5, 10, 15, 20, 25, and 30. In addition to the total RRS score, we examined: (1) two subscale scores (Brooding and Reflection subscales), (2) an RRS short-form score that combines the two subscales, as well as (3) a third subscale score consisting of the remaining questions on the full questionnaire not included on the RRS short form. The Brooding and Reflection scores each consisted of five RRS questions that gauge the tendency to focus on obstacles (Brooding) or to self-reflect (Reflection) [20], with the RRS short form score consisting of the sum of the Brooding and Reflection scores. The remaining 12 questions on the RRS that do not gauge Brooding or Reflection constituted an RRS Depressive Rumination score.

### rTMS procedure

rTMS treatment was administered using the Magstim Super Rapid Plus 1 stimulator (Magstim, Whitland, South Wales, UK), MagPro X100 (Magventure, Farnum, Denmark), or the Neuronetics Neurostar treatment system (Neuronetics, Malvern, PA, USA). Resting motor threshold (MT), defined as the minimum stimulation intensity necessary to elicit a detectable hand movement in ≥50% of single pulse trials, was performed prior to the first treatment [21]. In their initial treatment session, each patient received 3000 pulses 10 Hz stimulation to left dorsolateral prefrontal cortex (DLPFC) as determined by the Beam F3 method [22], administered in t 40-pulse trains with a 26s inter-train interva, and a maximum intensity of 120% MT. Subjects were treated under a measurement-based care paradigm in which stimulation parameters could be altered after the 10th treatment session for those individuals who failed to show benefit from the initial treatment parameters or had difficulty tolerating the procedure. Changes could include augmentation of stimulation (using theta burst priming or sequential bilateral stimulation) or changes to alternate stimulation sites or parameters (i.e., low-frequency right-sided stimulation) as described previously [23–27]. Because of the heterogeneity in the timing and nature of these changes, the stimulation protocol could not be included as independent group variables in our analyses. Instead, analyses of treatment outcomes are presented for the subjects overall.

### Statistical analyses

All statistical analyses were completed using RStudio v1.3.1093 and SPSS v27.0.0. Chi-squared tests were conducted to assess for differences in gender distributions and the ratio of treatment responders between groups. One-sample *t*-tests or Wilcoxon signed rank tests were conducted across the whole cohort to determine whether RRS and PHQ-9 scores significantly changed between the baseline and final treatment sessions.

To quantify depression treatment outcomes, we calculated PHQ-9 percentage change by dividing the score change between the final and baseline visits by the baseline PHQ-9 score for each individual. We repeated this method with the RRS total score, its three subscales, and the RRS short form score to determine the percentage change for all scales. Treatment response was defined as ≥50% improvement in PHQ-9. Outliers with a treatment outcome more than three standard deviations above the mean were excluded.

#### Association between depression and rumination

Pearson’s correlations were used to examine the associations between (1) baseline rumination and baseline depression severity (baseline PHQ-9 vs. baseline RRS), (2) baseline depression severity and treatment outcome (defined as the percentage change in PHQ-9 between the final and baseline time points), and (3) treatment outcome and rumination change (defined as RRS total percentage change). To determine whether rumination changes were mediated by treatment outcome, we performed a mediation analysis with an independent variable of baseline rumination, a dependent variable of rumination change, and a mediator of treatment outcome.

#### Multilevel modeling of rumination and treatment outcome

We assessed the relationship between baseline rumination and TMS treatment outcome using a series of repeated measures linear mixed-effects models (multilevel models), with PHQ-9 as the outcome variable and treatment time point and RRS score at baseline as fixed effects (Table 1a). We tested several nested models to assess whether adding additional variables, such as demographic variables and quadratic terms, would yield better model fits. Age and gender were included as fixed effects because of evidence that the efficacy of rTMS for treatment-resistant MDD patients may be influenced by age [28] and/or gender [29–31]. We explored the interaction between rumination and gender, and the interaction between rumination and time point (Model 4, Table 1a), and statistically compared baseline PHQ-9 and RRS total scores between genders.

**Table 1:** Summary of multilevel models.

a. Summary of multilevel models examining relationships among rumination and depression severity, demographics, and treatment outcome.^a^				
	Model 1	Model 2	Model 3	Model 4
Description	Base model	Inclusion of quadratic term	Inclusion of demographic covariates	Inclusion of demographic covariates and interactions
*Multilevel model*^b^				
	PHQ-9~RRS_baseline_ + Time Point	PHQ-9~RRS_baseline_ + Time Point + RRS_baseline_^2^	PHQ-9~RRS_baseline_ + Time Point + Age + Gender	PHQ-9~RRS_baseline_ + Time Point + Age + Gender + RRS*Gender + RRS*Time Point
Significant terms (at *p* < 0.05)	All	Time Point, RRS_baseline_^2^	RRS_baseline_, Time Point, Age	RRS_baseline_, Age, Gender
Statistics for RRS_baseline_	*t* = 11.01, estimate = 0.16, *p* < 0.001	*t* = −1.17, estimate = −0.14, *p* = 0.24	*t* = 10.57, estimate = 0.15, *p* < 0.001	*t* = 3.53, estimate = 0.14, *p* < 0.001
*Model comparisons*				
AIC^c^	6315.04	6310.96	6057.21	6066.60
BIC^c^	6342.43	6341.39	6090.69	6121.38
Model comparison^d^	–	Model 1 > Model 2	Model 1 < Model 3	Model 3 = Model 4
*p*-value	–	**0.01**	**<0.001**	0.72

b. Multilevel modeling of RRS subscales.^e^				
	Model 5	Model 6	Model 7	Model 8
Description	RRS short form	RRS Brooding	RRS Reflection	RRS Depressive Rumination
Multilevel model^b^	PHQ-9~RRS_baseline, short form_ + Time Point + Age + Gender	PHQ-9~RRS_baseline, Brooding_ + Time Point + Age + Gender	PHQ-9~RRS_baseline, Reflection_ + Time Point + Age + Gender	PHQ-9~RRS_baseline, Depression_ + Time Point + Age + Gender
Significant terms (*p* < 0.05)	RRS, Time Point, Gender, Age	RRS, Time Point, Gender, Age	RRS, Time Point, Gender, Age	RRS, Time Point, Age
Statistics for RRS term	*t* = 5.60, estimate = 0.09, *p* < 0.001	*t* = 5.24, estimate = 0.16, *p* < 0.001	*t* = 4.48, estimate = 0.14, *p* < 0.001	*t* = 5.41, estimate−0.08, *p* < 0.001
*Model comparisons*^f^				
AIC^c^	6138.73	6136.68	6143.98	6134.86
BIC^c^	6172.20	6170.16	6177.45	6168.34

*RRS* Rumination Responses Scale, *PHQ-9* Patient Health Questionnaire, depression scale.

^a^A comparison of two sets of nested multilevel models is shown. Upon direct statistic comparison of the two sets of models, Model 3 was chosen to be the best-fitting model for our data.

^b^For all models listed, the random effect of 1 + (Time Point|Subject) is included. Only fixed effects are shown in the table.

^c^Akaike’s Information Criteria (AIC) and Bayesian Information Criteria (BIC) are two common measures used to assess model fit, with smaller values indicating better fit.

^d^Models were directly compared using Likelihood Ratio Tests.

^e^A summary comparison of models including the RRS subscales (Brooding, Reflection, Depressive Rumination) is shown. Upon comparison of all models, the base model which includes the complete RRS scale fared best, underscoring the utility of the complete questionnaire.

^f^No direct model comparisons were made because the models were not nested.

*P*-values do not indicate whether an individual model is significant, but whether the two compared models are different. Bold values indicate a statistically significant difference between the compared models (at a threshold of *p* < 0.05).

Because a Loess fit [32] between PHQ-9 percentage change and baseline RRS suggested that the relationship might be non-linear, we also tested a model with a quadratic RRS term (Model 2, Table 1a). We used Akaike’s Information Criteria (AIC) and Bayesian Information Criteria (BIC) as measures of model fit, with lower values indicating better fits. To determine the best model to represent our data, we also directly compared nested models using the Likelihood Ratio Test. If two nested models performed similarly, we chose the more parsimonious model to represent our data.

We also assessed whether the RRS subscales yielded better model fits compared to the RRS total score by running four additional models, each with one of the RRS subscale scores in place of the RRS total score (Table 1b). We used AIC and BIC as indicators of model fit.

**Fig. 1:**
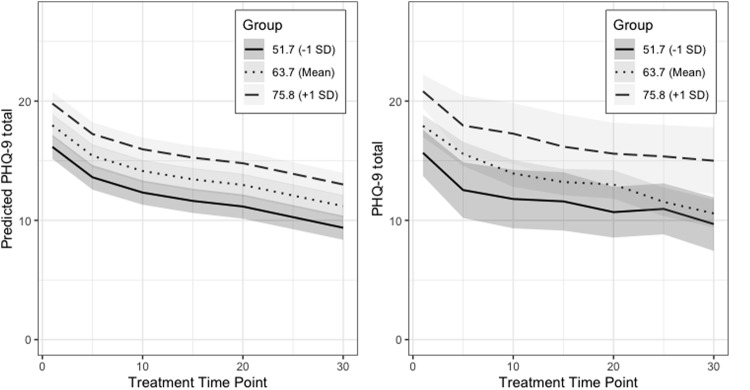
Estimated marginal means of PHQ-9 across treatments. Predicted values of PHQ-9 at each treatment time point were generated based on our final repeated linear mixed effects model (Table 1). The predicted PHQ-9 values in the left plot show the mean response of PHQ-9 at three representative RRS values, adjusted for other covariates in the model. From this model, PHQ-9 scores show a similar decrease over time at low (−1 SD), middle (mean), and high (+1 SD) values. The trajectories of real PHQ-9 scores over time are shown (right) grouped by those whose baseline RRS score fell within 1 standard deviation (SD) of the mean (63.7), and those whose baseline RRS score fell above or below 1 SD of the mean. The upper and lower limits of a 95% confidence interval are denoted with the shaded regions.

Finally, to assess how similar our predicted treatment trajectories were to our actual data, we statistically modeled the predicted PHQ-9 scores for each time point for multiple levels of our predictor variable, the RRS total score, and visually compared the trajectories to the actual treatment outcome trajectories in our cohort (Fig. 1).

## Results

### Patient demographics

rTMS treatment was associated with significant reductions in depressive and ruminative symptoms, as indicated by decreased scores for PHQ-9, RRS total, Brooding, Reflection, Depressive Rumination, and RRS short form (*p* < 0.001 for all). There were no statistically significant age or gender differences between depression responders and non-responders (Table 2). Depression responders and non-responders did not differ in any baseline measure of RRS or PHQ-9, but responders showed significant decreases in all measures of RRS and PHQ-9 after 30 treatment sessions compared to non-responders (Table 2). Age at first treatment was not associated with any measure of the RRS, PHQ-9, or depression treatment outcome.

**Table 2:** Patient demographics and measures across the whole cohort.

	All MDD patients	Depression responders	Depression non-responders	*p*-value^a^
*n*	155	58	97	–
Age	42.52 (14.22)	43.80 (14.93)	41.78 (13.83)	0.41
Gender (M:F)	76:79	27:31	49:48	0.76
RRS total, Tx 1	63.55 (12.00)	62.88 (11.97)	63.96 (12.06)	0.60
RRS total, Tx 30	50.06 (13.83)	41.17 (12.10)	54.77 (12.65)	**<0.001**
RRS total, % change	−20.81 (19.75)	−32.36 (17.69)	−13.77 (17.57)	**<0.001**
Brooding, Tx 1	14.75 (6.37)	14.13 (3.65)	15.13 (7.56)	0.28
Brooding, Tx 30	11.32 (4.93)	9.36 (3.13)	12.48 (5.43)	**<0.001**
Brooding, % change	−19.47 (31.93)	−32.00 (21.21)	−11.85 (34.93)	**<0.001**
Reflection, Tx 1	13.00 (6.02)	12.52 (2.86)	13.29 (7.31)	0.36
Reflection, Tx 30	9.85 (4.12)	8.97 (3.18)	10.37 (4.52)	**0.02**
Reflection, % change	−20.84 (26.21)	−27.81 (22.40)	−16.61 (27.54)	**0.008**
Depressive Rumination, Tx 1	35.79 (12.43)	36.23 (7.04)	35.53 (14.81)	0.70
Depressive Rumination, Tx 30	28.90 (9.92)	23.84 (7.30)	31.92 (10.08)	**<0.001**
Depressive Rumination, % change	−20.68 (24.69)	−33.66 (18.43)	−12.78 (24.76)	**<0.001**
RRS short form, Tx1	27.75 (12.01)	26.64 (5.76)	28.42 (14.54)	0.30
RRS short form, Tx 30	21.16 (8.50)	18.33 (5.83)	22.86 (9.38)	**<0.001**
RRS short form, % change	−20.69 (26.30)	−30.42 (19.69)	−14.76 (28.09)	**<0.001**
PHQ-9 total, Tx 1	17.99 (4.90)	18.00 (4.64)	17.98 (5.07)	0.98
PHQ-9 total, Tx 30	11.23 (6.08)	5.79 (2.98)	14.47 (5.06)	**<0.001**
PHQ-9, % change	−34.79 (41.53)	−67.50 (14.32)	−15.24 (40.15)	**<0.001**

Values show mean (SD). Changes are calculated by Tx 30–Tx 1. Tx: Treatment.

^a^Statistical comparisons were made between responders and non-responders.

Bold values indicate statistically significant *p*-values (*p* < 0.05).

#### Relationships between rumination and depression across all patients

**Fig. 2:**
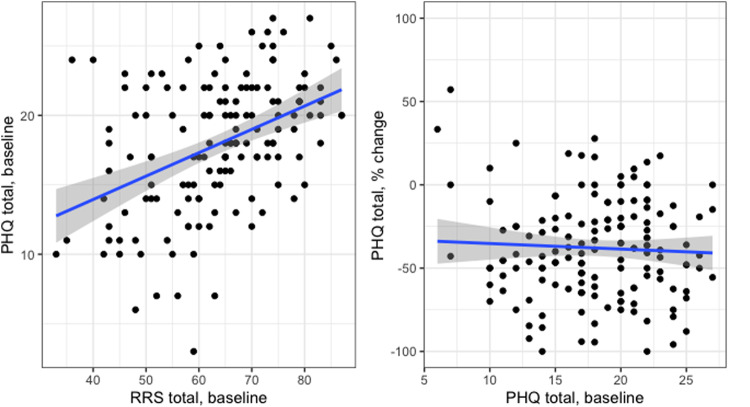
Baseline relationships between rumination, depression severity, and depression treatment outcome. Higher baseline rumination was significantly associated with more severe baseline depression (left). Baseline depression, however, was not a significant predictor of rTMS treatment outcome (right). A negative percentage change indicates an improvement in depression severity from baseline.

Baseline PHQ-9 was correlated with baseline RRS total (Fig. 2; *r* = 0.42, *p* < 0.001), Brooding (*r* = 0.17, *p* = 0.03), Depressive Rumination (*r* = 0.24, *p* = 0.003), and the RRS short form (*r* = 0.17, *p* = 0.04). Baseline PHQ-9 score also had a trend-level association with Reflection (*r* = 0.16, *p* = 0.06) scores. Baseline depression did not predict rTMS treatment outcome (Fig. 2). While depression improvement was correlated with rumination improvement after rTMS treatment (*r* = 0.57, *p* < 0.001), treatment outcome did not mediate the change in rumination (average mediation effect = 0.05, *p* = 0.5).

#### Modeling the relationship between rumination and depression

There was a significant linear relationship between RRS total score at baseline and treatment outcome; higher RRS was associated with poorer treatment outcomes (Model 1, Table 1a). The model including age and gender (Model 3, Table 1a) performed similarly to the base model (Model 1, Table 1a) and there were no significant interactions between RRS and gender, or between RRS and time points. Lower age was associated with worse treatment outcomes (Models 3–8, Table 1). We chose Model 3 as the best-fitting model for our data given that it was the most parsimonious model that included demographic variables previously reported to be associated with rTMS treatment outcomes [28–31]. Predicted treatment outcome trajectories from our chosen model were similar across low, middle, and high values of RRS, and trended similarly to actual treatment outcome trajectories, suggesting that the chosen model was a good representation of the data (Fig. 1).

#### Brooding, reflection, and depressive rumination

We also modeled the relationship between TMS treatment outcomes and three different subscales of the RRS, as well as a combined RRS short-form score. Higher RRS scores were significantly associated with worse treatment outcomes across all subscales (Table 1b). Gender was significantly associated with treatment outcomes when the RRS Brooding, Reflection, and short-form subscales were used (Table 1b), with women showing worse treatment outcomes compared to men. At baseline, women had higher PHQ-9 and RRS scores than men, although associations with gender did not persist after covarying for baseline RRS total score (Model 3, Tables 1a and 1b). Our base model which includes the RRS total score (Model 3) had the lowest AIC and BIC values across the five models, indicating the best fit (Table 1b).

## Discussion

The present results show that higher baseline rumination, but not depression levels, were associated with worse depression outcomes from rTMS treatment. The negative effect of rumination was stronger in female subjects. Improvement in depression and rumination were correlated, but reduction in rumination severity was not mediated by improvement in depression. These findings suggest that both rumination and depression symptoms were responsive to rTMS treatment, but that these were in part independent processes.

While there was a strong correlation between pretreatment severity of depression and rumination, the two baseline symptom measures differed in their relationships with overall depression treatment outcomes. Baseline depression severity did not predict treatment outcome, but a higher RRS total score at baseline was significantly associated with worse rTMS treatment outcomes, even after accounting for the potential effects of age and gender. Gender was a significant predictor of rTMS treatment outcome only when we modeled our data with the RRS Brooding, Reflection, and Depressive Rumination subscales, as well as the RRS short-form score.

Both depression and rumination symptoms appeared to be responsive to rTMS: there were significant decreases in the severity of both depression and ruminative thinking, although rumination decreased to a lesser extent (−20.5%) than depression (−31.6%). Given that this was not a controlled treatment study, these results do not prove a causal link between rTMS treatment and rumination improvement. There are, however, several key findings from the present study regarding the relationships between rumination and depression symptoms during rTMS treatment. First, baseline rumination levels were associated with the degree of antidepressant benefit from rTMS, indicating that rumination severity influenced the outcome of rTMS treatment. Second, while changes in rumination and depression severity were correlated, the antidepressant benefit of rTMS treatment did not mediate rumination improvement. This finding indicates that while the two variables were linked, depression change does not fully account for the improvement in rumination. Future double-blind and sham-controlled experimental studies should aim to elucidate the direct effects of rTMS on rumination.

It is important to note that the relationship between rTMS treatment outcomes and rumination levels was best modeled using the RRS total score. RRS subscales such as Brooding and Reflection captured rumination symptoms without confounding items which also measured depression, but using the RRS total score to modeled treatment outcomes yielding the best-fitting model [20]. The Reflection subscale of the RRS, however, was the least likely of the three subscales to be associated with depression and had the poorest model fit of all RRS subscales [20] (Table 1b). A recent study that utilized machine learning algorithms to find clinical, biological, and sociodemographic variables associated with rumination found that clinical scales of depression best-predicted rumination levels, in particular the Brooding subscale of the RRS, independent of psychiatric diagnosis [33]. Although the two RRS subscales had clinical and predictive utility, our finding that the RRS total score better-modeled rTMS treatment outcomes compared to the two subscales highlights the usefulness of the RRS total score when assessing rumination. Future studies can determine whether there are certain combinations of questions on the RRS outside of the Brooding and Reflection subscales which can predict rTMS treatment outcome better than the RRS total score.

Our findings corroborate previous literature on rumination and depression severity. Higher rumination has been associated with more severe depression [5, 12]. Previous studies of the RRS have reported that healthy individuals had mean scores ranging between 29 and 40 [34–38], while MDD subjects had mean scores ranging between 51 and 65 [34–36, 38, 39]. Our present cohort of MDD patients scored an average of 64 on the RRS, indicating that while our patients had more severe depression than previous cohorts, the severity of their rumination was at the high end of the range previously reported for other MDD cohorts. As such, in terms of rumination levels, our cohort can be considered comparable to other MDD cohorts, suggesting that our findings may be generalizable across the population of MDD patients.

Also consistent with prior literature, we found that the women had more severe depression and rumination at baseline compared to men. Women are at twice the risk of developing depression over their lifetime compared to men and tend to have longer and more severe episodes [40, 41]. A meta-analysis of gender differences in rumination revealed that women were more likely to ruminate compared to men [4]. Our results also indicate that high baseline rumination is implicated in rTMS treatment resistance, with higher baseline rumination scores associated with poorer rTMS outcomes even after accounting for the effects of age and gender. Previous studies have reported gender differences in rTMS treatment response rates, though the literature is mixed: one study found similar efficacy of rTMS treatment in men and women, although several later studies found that women achieved better outcomes [29–31]. To our knowledge, no previous study has explored how gender differences in rumination symptomatology affect rTMS treatment for depression. Although men and women in this study had similar rTMS depression treatment outcomes, when we modeled outcomes using the RRS short form as well as the three RRS subscales, we found that women with higher ruminative scores showed worse treatment outcomes, suggesting that ruminative symptoms may impede rTMS depression treatment efficacy.

Our findings are consistent with prior literature indicating that rumination symptoms can be ameliorated with treatment, although most previous studies have examined mild to moderate depression and not the more severe, treatment-refractory patients we examined here. These previous reports indicated that ruminations were poorly responsive to antidepressant medication treatment [7, 10]. In a small cohort of severely depressed patients, subanesthetic ketamine injections reduced RRS total scores from a median of 61–52 in a similar range to the score reduction we see with rTMS treatment [8]. Behavioral interventions specifically targeting rumination have been shown to have a larger effect than depression-focused treatments alone in reducing rumination, suggesting that perhaps new rTMS protocols can be developed to further reduce rumination in our cohort [3, 42]. The fact that ruminations were responsive to rTMS treatment is encouraging, and future studies should compare the efficacy of medications and/or behavioral interventions to that of rTMS in this population.

Another study by Kazemi and colleagues in 61 patients also demonstrated significant reductions in rumination and depressive symptoms after 20 rTMS treatments for depression [15]. This earlier study focused on comparing the effect of unilateral, bilateral, and sham stimulation to left DLPFC on rumination in a controlled-treatment trial and found reductions in rumination severity following treatment were significantly greater than in the sham group. However, Kazemi and colleagues did not examine the effects of baseline rumination severity or interaction between depressive and ruminative symptom improvement, and no information on the degree of treatment resistance or the influence of demographic factors was presented. While the current study was not a controlled treatment trial, the findings presented here represent an important replication and extension of earlier findings. Our sample size was 2.5 times larger and included a highly treatment-refractory MDD population, and demonstrate for the first time the effect of baseline rumination severity on rTMS antidepressant treatment outcomes. While the current study cannot establish a causal link between improvement in rumination and rTMS treatment, the mediation analysis does demonstrate that improvement in rumination cannot be fully accounted for by the improvement in depression. Furthermore, the current study has a high ecological validity of a measurement-based approach to rTMS treatment. The fact that efficacy for ruminations now has been seen in both a controlled and naturalistic study suggests a robust relationship that should be further examined using phenotyping and prospective treatment assignment studies. The consistency in evidence is particularly encouraging given the documented replicability crisis in psychiatry and neuroscience research [43–46].

There are several possible brain network mechanisms through which rumination may negatively impact rTMS treatment outcomes. rTMS treatment for depression usually targets the left DLPFC, a region typically responsible for cognitively demanding tasks and working memory [47]. The DLPFC is also a region within the central executive network, a brain network with correlated activity that is active during executive functioning [48]. Lowered activity within the central executive network has been observed during depression [49]. Rumination similarly recruits these same regions, although in the opposite direction; tasks that induce rumination increase activity within the DLPFC and central executive network [47]. It may be possible that very high levels of rumination alter brain network activity within the central executive network enough to impair the beneficial effects of rTMS treatment for depression, although further studies are needed to determine whether those who switched stimulation sites to other brain regions had a differing relationship between treatment outcome and rumination.

In addition, high rumination is associated with hyperactivity within the default mode network (DMN), a group of regions active during wakeful rest and inward-focused mental states [35, 50, 51]. The process of rumination activates the DMN during depression: regions of the DMN were active during ruminative thought in both adults and adolescents with a history of depression [52, 53]. Altered connectivity within the DMN is implicated across a range of psychiatric disorders, not only in MDD [1, 2, 54, 55], but also in bipolar disorder and schizophrenia [56, 57]. Hyperactivity within this shared network may be one explanatory factor for why rumination is a transdiagnostic risk factor that lends vulnerability across a range of psychiatric disorders. A recent rTMS study found that alterations in the DMN were associated with rumination score changes after rTMS treatment [15]. Future studies should further incorporate functional imaging measures to examine the mechanisms underlying the decreased rumination with rTMS treatment. Additionally, future studies are needed to discern how separate the effects of rTMS on rumination are from the effects of rTMS on depression.

The fact that improvement in rumination was not mediated by antidepressant benefit suggests that ruminative symptoms may need to be addressed specifically to maximize the benefit that patients experience from rTMS treatment. rTMS protocols may need to be tailored based on gender and rumination severity, potentially involving the addition of alternative sites of stimulation, to maximize treatment benefits for all patients. Potential stimulation sites to target rumination would include regions within the DMN accessible by rTMS, such as the medial prefrontal cortex or the inferior parietal lobule. Changes to other stimulation parameters to specifically decrease DMN activity may also prove to be effective for specifically altering rumination, although future experimental studies are needed. Further studies are also needed to explore exactly which dimensions of ruminative symptomatology beyond Brooding, Reflection, and Depressive Rumination may predict treatment outcomes in women and men. Additionally, future studies examine whether combining rTMS with behavioral interventions would be effective in reducing rumination in this treatment-resistant population.

These findings should be interpreted in the context of several limitations. First, this was not a controlled treatment study. Subjects were drawn from the population referred for rTMS treatment of MDD. We included only those subjects with more severe treatment-resistant illnesses but did not control for other aspects of clinical history. Some of these other factors could have influenced the results presented here. Second, all subjects initiated rTMS treatment with left DLPFC stimulation, but treatment after the 10th session could be modified under a measurement-based care paradigm with the addition of sequential stimulation targets or different frequencies. Because treatment parameters were modified based on response and tolerability rather than random assignment, it is not possible to examine the effect of specific treatment parameters on outcome. Third, subjects in our cohort tended to have more severe and refractory depression compared to MDD patients at large, although their rumination levels were comparable to other MDD cohorts. Future studies should examine whether rumination levels affect rTMS treatment outcomes differently in those with mild to moderate rather than severe depression. Fourth, subjects in this study received concomitant psychotropic medication treatment for MDD. It is possible that medication effects may have contributed to the findings reported here.

## Conclusion

Our results indicate that rumination as well as depressive symptoms are responsive to rTMS treatment, and that rumination symptoms negatively impact rTMS treatment outcomes for severely depressed and treatment-resistant patients. While rumination symptoms were responsive to rTMS treatment, they were less responsive than depressive symptoms, suggesting that specific treatment for ruminations may be useful in addition to conventional rTMS antidepressant paradigms. These findings underscore the importance of assessing rumination levels before rTMS treatment and highlight the need to better understand the underlying physiological mechanisms of rTMS effects on rumination. Future research should consistently examine the effects of baseline symptom severity, gender, age, and other clinical and demographic factors on treatment outcomes for not only depression but a range of other psychiatric disorders associated with high rumination.
